# Conotoxin Interactions with α9α10-nAChRs: Is the α9α10-Nicotinic Acetylcholine Receptor an Important Therapeutic Target for Pain Management?

**DOI:** 10.3390/toxins7103916

**Published:** 2015-09-28

**Authors:** Sarasa A. Mohammadi, MacDonald J. Christie

**Affiliations:** Discipline of Pharmacology, the University of Sydney, Sydney, NSW 2006, Australia; E-Mail: sarasa.mohammadi@sydney.edu.au

**Keywords:** α-conotoxins, α9α10-nicotinic acetylcholine receptors, pain

## Abstract

The α9α10-nicotinic acetylcholine receptor (nAChR) has been implicated in pain and has been proposed to be a novel target for analgesics. However, the evidence to support the involvement of the α9α10-nAChR in pain is conflicted. This receptor was first implicated in pain with the characterisation of conotoxin Vc1.1, which is highly selective for α9α10-nAChRs and is an efficacious analgesic in chronic pain models with restorative capacities and no reported side effects. Numerous other analgesic conotoxin and non-conotoxin molecules have been subsequently characterised that also inhibit α9α10-nAChRs. However, there is evidence that α9α10-nAChR inhibition is neither necessary nor sufficient for analgesia. α9α10-nAChR-inhibiting analogues of Vc1.1 have no analgesic effects. Genetically-modified α9-nAChR knockout mice have a phenotype that is markedly different from the analgesic profile of Vc1.1 and similar conotoxins, suggesting that the conotoxin effects are largely independent of α9α10-nAChRs. Furthermore, an alternative mechanism of analgesia by Vc1.1 and other similar conotoxins involving non-canonical coupling of GABA_B_ receptors to voltage-gated calcium channels is known. Additional incongruities regarding α9α10-nAChRs in analgesia are discussed. A more comprehensive characterisation of the role of α9α10-nAChRs in pain is crucial for understanding the analgesic action of conotoxins and for improved drug design.

## 1. Introduction

Pain is the emotional and sensory response to actual or potential tissue damage [1] and is vital for avoiding harm and for preventing further damage when recuperating from injury. In cases of chronic diseases or poor recovery from injury, this subjective pain experience becomes persistent. This transition from acute to chronic pain is incompletely understood, involving complex central nervous system (CNS) alterations, known as central sensitisation, that lead to pain hypersensitivity.

Chronic pain has conservatively been estimated to have a global prevalence of 22% [2]. Such estimates are likely to be underestimates due to the traditional view of pain as a secondary symptom to a primary disease; thus, primary diagnoses of pain are rare [3]. The common physiological and anatomical changes that occur in chronic pain sufferers have prompted some to argue for chronic pain to be considered as a disease entity in its own right [4,5]. In addition to the emotional and physical impact of chronic pain, the global economic burden is estimated to be in the hundreds of billions of dollars annually; a summation of costs to patients, carers, healthcare systems and the economy [3,5].

Currently available analgesics act via a limited number of molecular mechanisms. Chronic pain and neuropathic pain, which results from nerve injury or disease, are notoriously refractory to these pharmacological treatments. For mild to moderate pain, non-opioid analgesics, such as COX-inhibitors, are the primary means of treatment. However, these are inadequate in treating many neuropathic and chronic pain conditions and suffer from both ceiling effects and unfavourable side effect profiles [6]. Opiates are the most effective analgesics for acute pain, but are less effective for chronic neuropathic pain and are associated with significant adverse, dose-limiting side effects. Pregabalin and gabapentin were developed as anticonvulsants, but are now the first line treatment for some neuropathic pain conditions [7,8]. These drugs have high withdrawal rates due to the high risk of adverse events and are effective only in a minority of patients [8].

The narrow mechanistic range of current analgesic treatments can cause a patient’s treatment options to be rapidly exhausted, and patients are often left to endure chronic pain with only limited relief. Thus, in order to offer a broader range of treatment options to pain sufferers, new targets are being studied, including ion channels (e.g., calcium [9] and sodium channels [10]), transduction molecules (e.g., transient receptor potential (TRP) proteins [11,12,13]) and nicotinic acetylcholine receptors (nAChRs). Of great value to the study of such targets are conotoxins.

Conotoxins are peptides from the venoms of marine cone snails. Many conotoxins exhibit inherent selectivity and potency at mammalian cellular proteins, such as those involved in pain, and can therefore be used to better characterise those targets, while also holding potential as novel therapeutics themselves [14]. Heterogeneity in the subunit composition of certain ion channels, such as *N*-type calcium channels [15,16], sodium channels [17] and nAChRs [18], produces extensive structural diversity, which is recognised by many conotoxins. Thus, conotoxins offer great appeal as prospective selective therapeutics, with the potential of minimising off-target side effects.

Conotoxin peptides are classed according to their structure and respective ion channel or receptor target. Numerous conotoxin classes act on pain targets. Those classes of interest as potential analgesics include μ- and μO-conotoxins, which target voltage-gated sodium channels, ω-conotoxins, which target voltage-gated calcium channels, and α-conotoxins, which target nAChRs [19,20]. This review focuses on nAChR-mediated mechanisms of pain and the potential mechanisms of pain relief produced by α-conotoxins that interact with α9α10-nAChRs.

## 2. nAChRs Involved in Pain

Nicotinic acetylcholine receptors (nAChRs) belong to the ligand-gated ion channel superfamily, which also includes GABA_A_, GABA_C_, glycine and 5-HT_3_ receptors [21]. Human neuronal-type nAChRs exhibit a highly diverse composition of homo- or hetero-pentamers. Receptors are comprised of various combinations and permutations of alpha (α2-α7, α9, α10) and beta (β2-β4) subunits. Heteromers of α and β subunits are most abundant, while α7 is the only subunit known to form functional homomers. α8 and α9 subunits have been shown to form homomers in heterologous expression systems [22,23], but not in native systems.

nAChRs have long been the target of analgesic research, with little success. nAChR agonists, such as nicotine and epibatidine produce analgesia, but have small therapeutic windows and prohibitive side effect liabilities owing to their lack of selectivity [24,25]. Attempts to isolate the subunits responsible for nicotinic analgesia have identified α4β2- and α7-subunit-containing receptors; however, these subunits are not exclusively responsible for nicotinic analgesia [26]. Additional subunits, such as α3, α5 and β3, are believed to comprise part of the nicotinic analgesic effect [27,28,29].

The main factor that has limited the success of nAChR ligands is their narrow therapeutic window, *i.e.*, inadequate clinical efficacy and/or high incidence of adverse events [30,31]. Since cholinergic communication and regulation is so ubiquitous in the mammalian system and the complexity of nAChRs so great, therapeutic nAChR ligands continue to pose a great challenge.

## 3. α-Conotoxins and Pain

All Conus species studied thus far contain a unique combination of α-conotoxins that act as nAChR ligands that are selective for neuronal-type over muscle-type receptors and that are subunit selective. α-Conotoxins usually act as competitive antagonists [32,33], although the novel conotoxin, MrIC from *Conus marmoreus*, which has no agonist activity itself, acts as a co-agonist with the positive allosteric modulator, PNU120596, at the endogenous α7-nAChR [34]. The therapeutic potential of this novel agonistic action of an α-conotoxin has not been explored. α-Conotoxins are small peptides, 12–19 amino acids in length, and are identified by their conserved CC-C-C cysteine pattern. These cysteines form two disulphide bonds with I–III, II–IV connectivity, resulting in a two-loop framework, with varying numbers of residues within each loop ([35]; see Table 1).

Although several hundreds of α-conotoxins are expressed by Conus species [36], the potential pain-relieving actions of less than ten have been characterized in any detail. Some of the most promising analgesic conotoxins to be studied to date are Vc1.1 and RgIA. Both of these peptides exhibit the characteristic cysteine pattern of α-conotoxins and are antagonists at nAChRs. Early publications suggested that Vc1.1 interacted with nAChRs containing the α3 subunit with either β2 or β4; however, the affinity at these subunits was too weak to account for its analgesic effects with IC_50_ of 4200 and 7300 nM, respectively, at α3β2 and α3β4 recombinant rat nAChRs [37]. Vc1.1 was subsequently found to most selectively inhibit the α9α10-nAChR, with nanomolar affinity (19 nM at recombinant rat nAChRs [38]). This high functional selectivity for the α9α10-nAChR may overcome the classical challenges for nAChR-therapeutics, wherein functional potency does not necessarily reflect the binding affinities of nAChR inhibitors [31,37]. Interestingly, both Vc1.1 and RgIA selectively inhibit the α9α10-nAChR [38,39], which is an evolutionarily divergent nAChR subtype, believed to be most similar to ancestral forms of nAChRs [32]. As with all peptide drugs, α-conotoxins face concerns of low stability and poor bioavailability, which limits their therapeutic potential. Efforts to increase both resistance to enzymatic degradation and structural stability of α-conotoxins have resulted in the successful backbone cyclisation of both Vc1.1 [40] and RgIA [41], increasing their oral bioavailability. Dicarba analogues of Vc1.1 [42] and RgIA [43] have also shown improved stability, as well as enhancing selectivity for their proposed analgesic targets. *In silico* studies have further elucidated the binding properties of RgIA at the α9α10-nAChR [44].

**Table 1:** Analgesic α-conotoxins with proposed dual mechanisms of action.

Snail	Conotoxin	Sequence	Target		Analgesic?
			nAChR	Other	
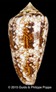	Vc1.1 *Conus victoriae*	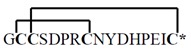	α9α10	*N*-type VGCC via GABA_B_R	Yes
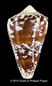	RgIA *Conus Regius*	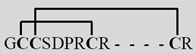	α9α10	*N*-type VGCC via GABA_B_R	Yes
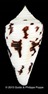	PeIA *Conus pergrandis*	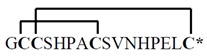	α9α10, α3β2	*N*-type VGCC via GABA_B_R	Not tested
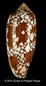	AuIB *Conus aulicus*	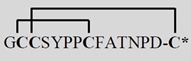	α3β4	*N*-type VGCC via GABA_B_R	Yes

* Amidated *C*-terminus. Lines linking the cysteine (C) residues in the conotoxin sequences represent disulphide bonds that contribute to the structural stability. VGCC, voltage-gated calcium channel. Images reproduced with permission © 2015 Guido and Philippe Poppe: www.conchology.be (accessed on 20 August 2015).

The α9α10-nAChR has very limited tissue distribution, with no known CNS expression or peripheral nervous system protein expression [22,45,46,47], but has a significant role in the cochlea and auditory system [22,48,49]. α-Conotoxins Vc1.1 and RgIA are highly effective analgesics in animal models of chronic pain [50], and this has implicated the α9α10-nAChR in pain for the first time. Although Vc1.1 began development for clinical use, it was dropped during phase IIa of clinical trials after its potency at human α9α10-nAChRs was found to be 100-fold lower than at rat α9α10-nAChRs [51]. This large inter-species difference in potency at the putative mechanistic target was deemed cost prohibitive (notified to the Australian Stock Exchange by Metabolic Pharmaceuticals Limited in 2007, [52]), and alternative α9α10-nAChR inhibitors continue to be sought.

The availability of α9α10-nAChR-selective conotoxins has been promoted as a bolster for pain-related research [32,53]. However, as discussed below, the precise role of the α9α10-nAChR in pain is now known to be complex, and the mechanism of action(s) of these analgesic α-conotoxins is not completely understood. Moreover, an alternative mechanism of action (discussed in Section 7) may account for many of the reported effects of Vc1.1 and RgIA. This alternative mechanism is likely shared by numerous other known conotoxins, such as PeIA and AuIB (Table 1), which may constitute a novel class of analgesics [54].

## 4. Evidence for α9α10-nAChR-Inhibition for Analgesia

As discussed below, the evidence to support the involvement of α9α10-nAChRs in pain comes solely from *in vivo* pharmacological studies.

### 4.1. Analgesic α-Conotoxins

Vc1.1 and RgIA have shown excellent analgesia in multiple rat models of chronic neuropathic pain. Intramuscular (i.m.) injection of these conotoxins has been shown to alleviate mechanical hyperalgesia [38,50,55] and mechanical allodynia [55,56,57] in models of chronic constriction injury (CCI) and partial nerve ligation (PNL) of the sciatic nerve. Intrathecal injection of Vc1.1 has also been shown to alleviate PNL-induced mechanical allodynia [58]. A cyclised version of Vc1.1 (cVc1.1) has shown anti-allodynic efficacy in CCI-induced neuropathic pain after oral administration [40]. Independent testing of Vc1.1 by Metabolic Pharmaceuticals Pty. Ltd. (now a subsidiary of PolyNovo Ltd, formerly Calzada Ltd, Melbourne, Australia) confirmed the anti-allodynic and anti-hyperalgesic effects of i.m. Vc1.1 in the PNL and CCI models, as well as observing analgesic efficacy of Vc1.1 in pain associated with diabetic neuropathy (streptozotocin model) and inflammatory pain (at the highest doses only; complete Freund’s adjuvant (CFA) model) (previously posted in the Metabolic Pharmaceuticals Limited information sheet as cited in [59]).

In addition to acute analgesia, Vc1.1 has been shown to have long-acting effects that last well after the peptide has cleared. Repeated daily dosing of Vc1.1 for seven days has cumulative effects, with analgesia persisting for at least one week after the cessation of treatment [50,60].

### 4.2. Functional Recovery

Vc1.1 has been reported to accelerate the functional recovery of injured peripheral nerves. Satkunanathan *et al.* [50] examined CCI-injured rats that had been treated for seven days with Vc1.1 during the course of neuropathic pain development, but had ceased conotoxin treatment approximately six weeks prior to functional testing. Blisters were raised on the glabrous skin of the injured hind limbs, and the peripheral vascular responses were monitored with laser Doppler flowmetry. When substance P, a potent vasodilator, was perfused over the blister in injured animals, the Vc1.1-treated animals exhibited a vascular response significantly closer to uninjured rats than the saline-treated animals. The relatively normal inflammatory vascular response to substance P in the Vc1.1-treated animals suggests that there was functional recovery in the previously injured nerves of the conotoxin-treated rats.

In a similar blister-induction model observing peripheral vascular responses, Sandall *et al.* [61] applied antidromic electrical stimulation of *C*-fibres in naive rats. Electrical stimulation of *C*-fibres induces vasodilation of the microvasculature in the blistered region. This was dose-dependently inhibited by Vc1.1 perfusion over the blister; thus, the peptide was postulated to act by reducing peripheral neurotransmitter release from the stimulated nociceptive *C*-fibres.

Whether the functional recovery observed in the Vc1.1-treated animals [50] and the acute Vc1.1-mediated inhibition of *C*-fibre neurotransmitter release [61] occur via the same mechanism is unknown. The unmodified, native form of the Vc1.1 peptide, Vc1.1ptm (also referred to as vc1a), similarly accelerates functional recovery [60] without producing significant analgesia [56,60]. This suggests that the functional recovery seen in α-conotoxin-treated animals may occur via mechanisms independent of the analgesic mechanisms. Whether these are α9α10-nAChR dependent is not known.

Histological changes in Vc1.1- and RgIA-treated animals have also been observed, wherein CCI-injured rats that are treated with these analgesic conotoxins show reductions in immune responses and injury markers. Vincler *et al.* [38] observed significant reductions in the infiltration of injured sciatic nerves by immune cells (CD2+ T-lymphocytes, CD68+ macrophages) and choline acetyltransferase positive (ChAT+) cells in Vc1.1 and RgIA-treated rats. More detailed histological investigations have revealed apparently neuroprotective effects of RgIA. Mannelli *et al.* [55] observed that after 14 days of daily RgIA administration, the number of fibres, myelin thickness, axon diameter, oedema, infiltrate, CD86+ and GFAP+ cells, nucleolus changes and glial cell changes are all significantly closer to those of sham animals than in injured, vehicle-treated animals. These effects were attributed to α9α10**-**nAChR inhibition. However, similar changes have not been observed in α9-nAChR knockout mice [62], suggesting that the conotoxin-mediated effects may be unrelated to inhibition of α9α10-nAChRs.

### 4.3. Side Effects

Analgesic α-conotoxins show promise as a novel class of analgesics that avoid many problematic adverse events that are associated with current analgesics, such as opiates. To date, no negative side effects have been reported in peer-reviewed publications after treatment with analgesic α9α10-inhibiting conotoxins, such as Vc1.1, RgIA and AuIB [63]. Metabolic Pharmaceuticals Pty. Ltd. performed safety profile analyses on Vc1.1 and found no effect on bodyweight, food consumption, ophthalmic parameters, haematology, blood chemistry, urinalysis, organ weights, macropathology, histopathology and no detectable immune response at any dose level in rats and mini-pigs. No motor effects (rat and mouse; Irwin test battery, accelerating rotarod) or respiratory effects (whole body plethysmography) were observed. Some cardiovascular effects were found at higher doses (dog telemetry; increased heart rate, decreased blood pressure) (previously posted in the Metabolic Pharmaceuticals Limited information sheet as cited in [59]).

Furthermore, no apparent tolerance has been observed with repeated dosing of Vc1.1; rather, there is a cumulative analgesic effect (Satkunanathan *et al.* [50], dose for seven days, test one week after final dose; Vincler *et al.* [38], dose for four days). Lack of tolerance suggests a useful alternative to opioid analgesics that are known to produce considerable tolerance with chronic treatment.

### 4.4. Non-Peptide, Small-Molecule α9α10-nAChR Inhibitors

Recently, several non-peptide, small-molecule α9α10-nAChR antagonists have been reported that have analgesic effects [64,65]. These compounds add further support to the possibility of an involvement of the α9α10-nAChR in pain. The quaternary ammonium analogues of nicotine were reported to achieve specific pharmacological block of the α9α10-nAChR. These compounds were found to be effective at attenuating the development of vincristine-induced, neuropathic pain (von Frey and paw pressure vocalisation threshold) and phase II formalin pain, as well as acutely relieving CCI and vincristine pain (paw pressure vocalisation threshold) at high doses [64,65].

However, as with the α-conotoxin studies, these assertions are only as reliable as the selectivity, pharmacokinetics and pharmacodynamics of the compounds used. One non-peptide small molecule, ZZ-204G [64], caused motor incoordination (rotarod) as a side effect at high doses, which has been shown not to occur in α-conotoxin studies (Metabolic Pharmaceuticals info sheet, as cited in [59]). This indicates that ZZ-204G also acts at non-α9α10-nAChR sites. Although these small-molecule nicotine analogues have been designed with high selectivity for the α9α10-nAChR, interactions with less common nAChR subunits or non-nAChR proteins is a possibility. Given the apparent lack of specificity, these nicotine analogues are unlikely to elucidate the role of α9α10-nAChRs in the *in vivo* context.

The dependence on pharmacological agents to characterize the functional role of receptor subtypes carries a significant risk of unknown functions of such compounds being misattributed to the known targets.

## 5. Evidence against α9α10-nAChR-Inhibition for Analgesia

Despite the promising results of the α-conotoxin studies, the mechanism behind the analgesic actions of α-conotoxins, such as Vc1.1 and RgIA, has not been confirmed. Assertions that the inhibition of α9α10-nAChRs is the mechanism of analgesia of these conotoxins are supported only by indirect evidence, and there is now sufficient evidence to rule out α9α10-nAChR inhibition as the primary analgesic mechanism of α-conotoxins. The analgesic activity of α9α10-nAChR-selective drugs is summarised in Table 2.

### 5.1. Vc1.1 Analogue and Native Peptide

Pharmacological evidence for the insufficiency of α9α10-nAChR inhibition for analgesia was first shown by Nevin *et al.* [56]. The authors showed that the native peptide and an analogue of Vc1.1 that both retained their potency at and selectivity for α9α10-nAChRs produced no analgesia (von Frey threshold) in the PNL model of neuropathic pain in rats. The native peptide, vc1a, and the analogue [P6O]Vc1.1, were structurally almost identical to Vc1.1 apart from one (for [P6O]Vc1.1) or two (for vc1a) post-translational modifications (PTMs), indicating that any differences in biological targets were not due to major changes in the 3D shape of the molecules. The inability of the modified Vc1.1 peptides to alleviate pain, despite their equipotency with Vc1.1 at the α9α10-nAChR, clearly indicates the insufficiency of α9α10-nAChR inhibition for analgesia.

### 5.2. α9-nAChR KO Phenotype

Behavioural phenotyping of mice that have a germline deletion of the α9-nAChR have recently uncovered a unique pain phenotype that is starkly mismatched with α-conotoxin analgesic effects. α9-nAChR knockout (KO) mice were found to have a largely normal pain phenotype with only a single pain modality showing alteration from wild-type (WT) animals [59]. Naive KO mice showed completely normal nociceptive responses in all pain modalities tested, including the von Frey test, paw pressure test, hotplate test and acetone test. Pain models of neuropathic (CCI) and inflammatory (CFA) pain revealed a normal phenotype with respect to most pain modalities, including mechanical allodynia (von Frey threshold), thermal hyperalgesia and cold allodynia. An altered pain phenotype was, however, observed for mechanical hyperalgesia. Both the development and maintenance of chronic mechanical hyperalgesia were attenuated in KO mice. This pain phenotype does not mirror the anti-allodynic effects that are seen in conotoxin analgesia, indicating that at least part of the analgesic effects of α-conotoxins occur via non-α9α10-nAChR mechanisms.

**Table 2:** Summary of the *in vivo* analgesic activity of α9α10-nAChR-selective drugs.

Compound Name	Analgesic?					Side Effects?	Functional Recovery?	References
	Nerve Injury (PNL or CCI)			Formalin	Vincristine			
	Von Frey	R-S	Incap.					
Vc1.1	Yes	Yes	-	-	-	No	Yes	[38,50,56,57,58,59,60]
RgIA	Yes	Yes	Yes	-	-	N/R	-	[38,55]
vc1a	No	-	-	-	-	N/R	Yes	[56,60]
[P6O]Vc1.1	No	-	-	-	-	N/R	-	[56]
cVc1.1	Yes	-	-	-	-	N/R	-	[40]
ZZ-204G	-	Yes	-	Yes	-	Yes	-	[64]
ZZ1-61c	-	-	-	-	Yes	No	-	[65]

All *in vivo* testing was performed in rats. Dashes indicate where no testing has been reported. CCI, chronic constriction injury; Incap., incapacitance test; N/R, none reported; PNL, partial nerve ligation; R-S, Randall-Selitto test.

## 6. The Site(s) of Action of α9α10-nAChR Inhibiting Analgesics is Unknown

### 6.1. Immune Cells

**Figure 1:**
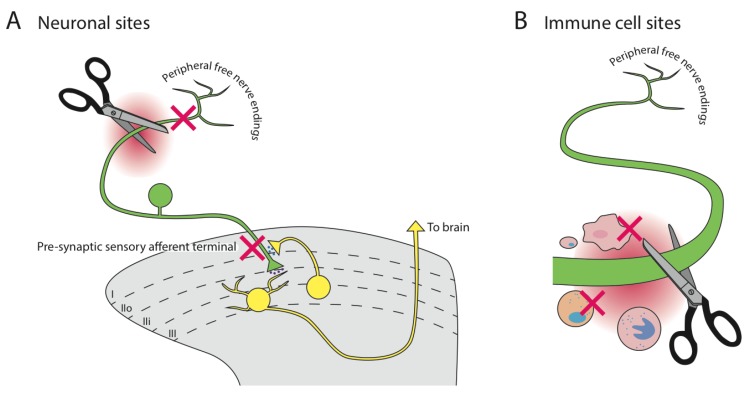
Proposed sites of action of analgesic α-conotoxins, such as Vc1.1. (**A**) Neuronal sites on peripheral sensory nerves (green) have been proposed, inhibiting (red Xs) either peripheral or central terminals. Central cholinergic neurons (yellow) are an ACh source. (**B**) Immune cell sites have been proposed. Scissors represent the sites of injury along the sensory afferent nerves.

The putative mechanism of analgesia of α9α10-nAChR antagonists is through inhibition of immune cells [38,55] (Figure 1B). Support for this comes from the finding that repeated Vc1.1 or RgIA administration in rats significantly inhibited the migration of ChAT-immunoreactive cells, ED1-immunoreactive macrophages and CD2-immuonreactive T-cells into CCI injured nerves [38]. The high degree of selectivity of these conotoxins for α9α10-nAChRs was inferred to be the mechanism of action of both the inhibition of immune cell infiltration and the analgesia. The authors suggested that inhibition of α9α10-nAChRs on immune cells in the vicinity of nerve injury reduces the inflammatory milieu, thus reducing the overall algogenic pathology. However, similar suppression of immune reactions to neuropathic injury models are not seen in α9-nAChR KO mice [62], suggesting that the α-conotoxin-mediated effects seen by Vincler *et al*. [38] and Mannelli *et al*. [55] are not α9α10-nAChR-dependent effects.

Peripheral immune cells do express the main components of cholinergic communication, including nAChRs, choline acetyltransferase (ChAT) and ACh [66,67,68], so they could feasibly be targets for nicotinic analgesics. However, the presence of functional, ACh-responsive α9α10-nAChRs on immune cells is yet to be confirmed, and as yet, attempts to elicit α9α10-mediated ACh responses have not succeeded in either human B- or T-lymphocytes or Jurkat immortalised T-cells [69].

There is therefore little doubt that Vc1.1 and RgIA (and perhaps Vc1a) inhibit the invasion of immune cells into injured nerves, and this may mediate some of their analgesic and recuperative effects. However, the proposal that inhibition of α9α10-nAChRs on these cells is the mechanism underlying this action is questionable.

### 6.2. Neuronal Cells

Another potential site of action of systemically-acting analgesics is the peripheral sensory nervous system (Figure 1A). Unfortunately, no functional expression of α9α10-nAChRs has been shown on sensory afferent nerve axons, terminals or cell soma. The cell bodies of peripheral sensory nerves, collectively situated in the dorsal root ganglia (DRGs), do indeed express multiple nAChR subtypes [70], though these are predominantly α4 and α7 [46,70]. α9-nAChR mRNA expression has been inconsistently found in rat DRG neurons; however, no translated functional protein has been detected [45,46,47]. Putting this mechanism of action into further doubt is the fact that nAChRs have been shown to be downregulated in peripheral sensory afferents in neuropathic pain models [71], and *in vivo* studies show that α-bungarotoxin (α-BGTx)-sensitive nAChR subtypes (*i.e.*, α7 and α9) are minimally involved in nicotinic analgesia [72]. Therefore, inhibition of α9α10-nAChRs on peripheral nerves is very unlikely to explain the analgesic actions of the α-conotoxins.

Studies that have investigated the action of conotoxins on peripheral nerve cells have generally tested responses in dissociated DRG cell bodies. In many of these studies, the tissue preparation process uses enzymatic dissociation processes that may render the α9α10-nAChR inactive. Collagenase, the primary digestive enzyme used, uncouples the α9α10-nAChR from small conductance Ca^2+^-dependent K^+^ channel (SK2), which is a complex that has been shown to be necessary for α9α10-nAChR receptor function [73,74]. However, it is possible that the requirement of α9α10-nAChR/SK2 coupling is specific to the cochlear and vestibular hair cell types, in which this phenomenon was characterized, as functional α9α10-nAChRs have been recombinantly expressed in *X. laevis* oocytes [37,38,40,56,58,75].

### 6.3. Acetylcholine Source

For peripherally-acting analgesic α-conotoxins to act via nAChR inhibition, an intrinsic ACh source must be present at injury sites. A peripheral origin of a cholinergic plexus has been suggested that could account for nAChR activation, but the evidence is conflicted. Both the absence [76] and presence [77] of ChAT-immunoreactive DRG cells have been reported with the same antibody. The functional role of ACh in peripheral sensory neurons is speculated to be central inhibition of pain [77], though more evidence is needed to support this theory. A sub-group of nociceptors (capsaicin-sensitive) do not release ACh centrally [78]. Whether other sub-groups of nociceptive fibres do release ACh centrally remains to be determined.

Non-neuronal ACh sources include keratinocytes after cutaneous injury [79], as well as immune cells. A cutaneous source would not account for the pain relief attained by Vc1.1 in animal models, which involve nerve injury (CCI, PNL [38,40,50,56,57]), inflammatory (CFA) and chemogenic (diabetic neuropathy via streptozotocin injection pain). Immune cells, such as lymphocytes, dendritic cells and macrophages, express cholinergic components sufficient to constitute a discrete cholinergic system, synthesising and releasing ACh that has either an autocrine or paracrine effect [66,80]. Whether or not ACh released from immune cells does activate sensory afferent nerve nAChRs is unknown. It is possible that the main function of such ACh sources is activation of immune cell nAChRs, as nAChRs mRNA has been identified in thymocytes (α3, α5, β4 [81]) and lymphocytes (α2, α5, α6, α7, α10, β2 [82]). α9α10-nAChR protein has been identified in B- and T-cells; however, these receptors were unresponsive to applied ACh [69].

## 7. An Alternative Mechanism of α-Conotoxin Analgesia Is Known

**Figure 2:**
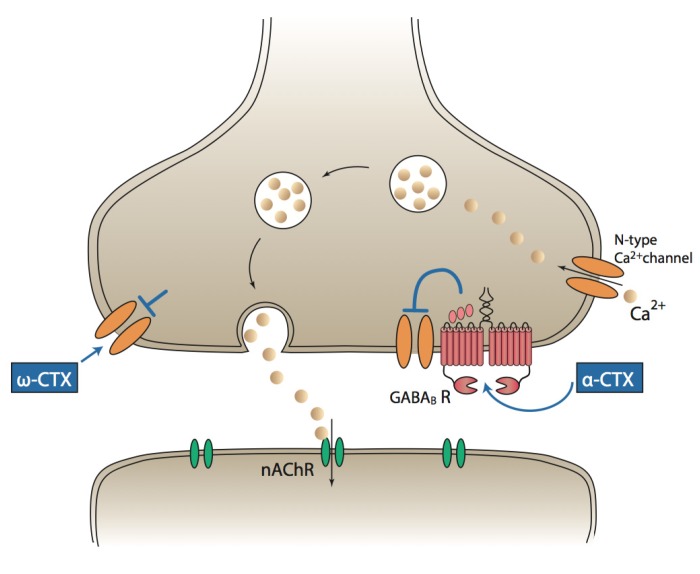
Putative mechanisms of action of VGCC-inhibiting conotoxins. α-Conotoxins (α-CTX), such as Vc1.1, are thought to bind to GABA_B_ receptors, which are coupled to *N*-type Ca^2+^ channels. Conotoxin binding indirectly prevents Ca^2+^ entry through these Ca^2+^ channels. ω-Conotoxins (ω-CTX) bind to Ca^2+^ channels and directly inhibit Ca^2+^ entry.

An alternative mechanism of action of α-conotoxin analgesia that does not involve nAChRs has been identified. α-Conotoxins, such as Vc1.1 and RgIA, potently and selectively inhibit the *N*-type component of high-voltage-activated (HVA) calcium channel currents in dissociated DRG neurons. This inhibition of *N*-type VGCC inhibition is dependent on GABA_B_R binding and completely independent of α9α10-nAChRs, but occurs via a non-canonical G-protein-mediated mechanism [45,83] (Figure 2). Inhibition of peripheral sensory nerve *N*-type VGCCs is thus believed to confer α-conotoxin analgesia via preventing the transmission of nociceptive input from the periphery to higher order centres. Further support for the GABA_B_R-dependent VGCC inhibition being the primary analgesic mechanism of α-conotoxins is the finding that Vc1.1-analogues that retain the α9α10-nAChR inhibitory properties, but not the VGCC inhibition [45], do not alleviate pain in animal models [56]. The importance of GABA_B_R binding for α-conotoxin analgesia has been confirmed *in vivo* through the co-administration of a GABA_B_R antagonist with Vc1.1 in rats, which completely abolished Vc1.1 analgesia [57].

Increasingly, more α-conotoxins with this unique GABA_B_R-dependent VGCC inhibiting mechanism are being identified that show promise as novel analgesics (Table 1). Other α-conotoxins have also been identified that possess this GABA_B_R-dependent VGCC inhibitory mechanism, such as PeIA and AuIB [84,85]. Although the nAChR targets of these other conotoxins vary (PeIA inhibits α9α10, α3β2, α6/α3β2β3 [86] and AuIB inhibits α3β4 [87]), the common VGCC inhibiting mechanism is proposed to confer analgesic properties to both [54,88]. AuIB has been shown to be analgesic, while PeIA remains to be tested [57,58]. The inhibition of their respective nicotinic subunits may also contribute to their analgesia; however, the diversity of nAChR-subtypes inhibited by this group of α-conotoxins suggests that the inhibition of VGCCs via GABA_B_Rs is likely to be the primary mechanism of analgesia.

## 8. Conclusions

The discovery of analgesic α9α10-nAChR-inhibiting conotoxins highlighted the role of the α9α10-nAChR in pain for the first time. The presence of at least two mechanisms of action of Vc1.1 and RgIA has likely masked the dissociation between α9α10-nAChR-specific effects and other mechanisms. Inhibition of the α9α10-nAChR may be conferring an additional attenuating and restorative capacity to the conotoxins, alongside the acute analgesic effects via GABA_B_-dependent *N*-type VGCC-inhibition [45,57]. *In vivo* α9-nAChR KO experiments suggest that the inhibition of the α9α10-nAChR may have been erroneously attributed to be the mechanism of acute α-conotoxin analgesia.

As with all pharmacological studies, the assertions of the involvement of this receptor in pain are only as reliable as the selectivity, pharmacokinetics and pharmacodynamics of the compounds used. There is a significant risk of unknown functions of compounds being misattributed to the known targets. The non-analgesic Vc1.1 analogues, the α9-nAChR KO studies and the characterization of an alternative mechanism of analgesia all support the notion that α9α10-nAChRs play a relatively minor role in pain perception and that conotoxins, such as Vc1.1 and RgIA, largely achieve their effects via α9α10-nAChR-independent mechanisms. While further characterization of the α9α10-nAChR in pain states is required, the evidence to date suggests that the involvement of the receptor in pain mechanisms and treatment has been overstated.
